# How fructophilic lactic acid bacteria may reduce the FODMAPs content in wheat-derived baked goods: a proof of concept

**DOI:** 10.1186/s12934-020-01438-6

**Published:** 2020-09-17

**Authors:** Marta Acín Albiac, Raffaella Di Cagno, Pasquale Filannino, Vincenzo Cantatore, Marco Gobbetti

**Affiliations:** 1 Faculty of Sciences and Technology, Libera Università di Bolzano, 39100 Bolzano, Italy; 2 Department of Soil, Plant and Food Science, University of Bari Aldo Moro, 70126 Bari, Italy

**Keywords:** FODMAP, IBS, Fructophilic lactic acid bacteria, Fructans, Wheat flour, Bread

## Abstract

**Background:**

FODMAPs (Fermentable oligosaccharides, disaccharides, monosaccharides, and polyols) intake is associated with the onset of irritable bowel syndrome symptoms. FODMAPs in wheat-derived baked goods may be reduced via bioprocessing by endogenous enzymes and/or microbial fermentation. Because of the inherent enzyme activities, bread made by baker’s yeast and sourdough may result in decreased levels of FODMAPs, whose values are, however, not enough low for people sensitive to FODMAPs.

**Results:**

Our study investigated the complementary capability of targeted commercial enzymes and metabolically strictly fructophilic lactic acid bacteria (FLAB) to hydrolyze fructans and deplete fructose during wheat dough fermentation. FLAB strains displayed higher fructose consumption rate compared to conventional sourdough lactic acid bacteria. Fructose metabolism by FLAB was faster than glucose. The catabolism of mannitol with the goal of its reuse by FLAB was also investigated. Under sourdough conditions, higher fructans breakdown occurred in FLAB inoculated doughs compared to conventional sourdough bacteria. Preliminary trials allowed selecting *Apilactobacillus kunkeei* B23I and *Fructobacillus fructosus* MBIII5 as starter candidates, which were successfully applied in synergy with commercial invertase for low FODMAPs baking.

**Conclusions:**

Results of this study clearly demonstrated the potential of selected strictly FLAB to strongly reduce FODMAPs in wheat dough, especially under liquid-dough and high oxygenation conditions.

## Background

Fermentable oligosaccharides, disaccharides, monosaccharides, and polyols (FODMAPs) are heterogeneous compounds, which, due to their chemical structure, are poorly digestible and may exert a range of effects on human gastrointestinal process. Fructans, galacto-oligosaccharides (GOS), lactose, fructose in excess of glucose, and sugar polyols (sorbitol and mannitol) are the main FODMAPs found in foods [1, 2]. Whether some FODMAPs are considered prebiotics contributing to the healthy maintenance of intestinal microbiota, on the other side, FODMAPs intake is associated with the onset of irritable bowel syndrome (IBS) symptoms and other functional gut disorders [1, 2]. IBS is the most common gastrointestinal disorder worldwide, with a prevalence estimated between 7 and 21% of the global population [2]. Triggering of IBS symptoms following consumption of FODMAPs is due to their poor absorption at the small intestine level. Some FODMAPs are osmotically active, and their malabsorption draws water into the intestinal and/or colonic lumen, causing diarrhea. Once reached the distal ileum and colon, FODMAPs undergo the fermentation to short-chain fatty acids and gases, causing bloating, abdominal pain, and flatulence [1, 2]. FODMAPs may also trigger the manifestation of non-celiac wheat sensitivity [3, 4]. A low-FODMAPs diet may reduce symptoms associated to IBS or non-celiac wheat sensitivity [5, 6]. Being FODMAPs responsible for diversified effects along the gastrointestinal tract, a cutoff value for each compound was definable, which ranges from 0.15 to 1 g per serving/meal for IBS patients [7]. Anyway, a diet therapy based on the avoidance of FODMAPs restricts too much the food choices, eliminating staple foods, likely wheat derivatives and various vegetables, legumes and fruits. Under this dietary regimen, IBS patients should suffer for reduced intakes of fiber, minerals, vitamins, and phytochemicals. In addition to the unbalanced nutritional status, the drastic reduction of FODMAPs interferes with the intestinal microbiota and colonocyte metabolism [8].

Wheat-derived baked goods account for the major part of the daily consumed FODMAPs, mainly represented by fructans and, to a lesser extent, GOS and fructose, whereas mannitol is mostly present in sourdough bread [9]. Considering the risks associated to prolonged implementation of low-FODMAP diet, various biotechnology strategies are under investigation for the manufacture of wheat-derived baked goods with reduced FODMAPs content and suitable for IBS patients [10]. For instance, the use enzymes degrading fructans lowered the level of FODMAPs in bread. Whether humans lack these enzymes, they are common in many plants and microorganisms. Exoinulinases (EC 3.2.1.80) are specific for the terminal linkages of inulin and release fructose, whereas endoinulinases (EC 3.2.1.7) have as target the internal β(2–1) glycosidic linkage, resulting into oligosaccharides with a lower degree of polymerization. Using an exo-mechanism, invertase (β-fructofuranoside fructohydrolase EC 3.2.1.26) is able to catalyze the degradation of inulin [10]. Apart from the enzyme used, the hydrolysis of fructans correlates with the release of fructose, which is also within the FODMAP group, but only when present in excess to glucose. Indeed, when fructose is present at the luminal level together with equimolar amounts of free glucose, the apical GLUT2 transporter prevents the risk of fructose malabsorption [11]. Because of the inherent enzyme activities, bread making by baker’s yeast and sourdough may result in decreased levels of FODMAPs, whose values are, however, not enough low for people sensitive to FODMAPs. Several causes might be responsible for this unsatisfactory result, likely the short leavening/fermentation time, the release of fructose from fructans, and the capability of hetero-fermentative lactic acid bacteria to convert the fructose to mannitol [10]. Therefore, low FODMAP baking needs dedicated approaches targeting fructans-, fructose-, and mannitol-degrading enzymes/organisms [9].

Our study aimed at investigating the complementary capability of targeted commercial enzymes and strictly fructophilic lactic acid bacteria (FLAB) to fully degrade FODMAPs during wheat dough fermentation. As a newly discovered bacterial group, FLAB are gaining increasing interest for their potential applications in food and pharmaceutical field [12]. First, we screened strictly FLAB for their fructose consumption rates and mannitol degrading capabilities. Besides, we developed a novel protocol applied both under firm- and liquid-dough fermentation [13].

## Results

### Fructose consumption rate by FLAB during growth into fructose-glucose based medium

Fructose consumption rate by FLAB strains, as well by *Lactiplantibacillus plantarum* DC400 (formerly *Lactobacillus plantarum*) and *Fructilactobacillus sanfranciscensis* SD8 (formerly *Lactobacillus sanfranciscensis*) (controls), was investigated during growth into Fructose Glucose Yeast extract Peptone (FGYP) broth under aerobic conditions. After 24 h at 30 °C, all the strains reached a cell density of ca. 9‒9.5 Log CFU ml^−1^. The fructose available in the medium (10 g L^−1^) was completely consumed after 12–14 h by all FLAB, with the exception of *Apilactobacillus kunkeei* PLB29 and PFA3 (formerly *Lactobacillus kunkeei*), and *Fructobacillus fructosus* PFA23, for which depletion was reached after 16 h. *L. plantarum* DC400 and *F. sanfranciscensis* SD8 slowly metabolized fructose; it was still detectable after 24 h. Kinetics of fructose consumption were modeled according to the Gompertz equation as modified by Zwietering et al. [14] (Table 1). *A. kunkeei* strains showed *μ*_*max*_ values ranging from 1.28 ± 0.18 to 3.51 ± 0.10 g L^−1^ h^−1^, where *λ* values ranged from 1.47 ± 0.17 to 8.32 ± 0.28 h. When *Fructo. fructosus* strains were used, *μ*_*max*_ and *λ* ranged from 0.96 ± 0.15 to 3.02 ± 0.15 g L^−1^ h^−1^, and from 3.07 ± 0.25 to 5.32 ± 0.32 h, respectively. As expected, the lowest (P < 0.05) values of *μ*_*max*_ were found with *L. plantarum* DC400 (0.28 ± 0.14 g L^−1^ h^−1^) and *F. sanfranciscensis* SD8 (0.88 ± 0.08 g L^−1^ h^−1^). This latter had also the highest value of *λ* (13.76 ± 0.39 h). On the other side, glucose metabolism by FLAB was slower than fructose (data not shown).

**Table 1:** Kinetics parameters (*μ*_*max*_ and *λ*)* of fructose consumption by bacterial strains

Strain	*μ*_*max*_	*λ*
*Apilactobacillus kunkeei* BIII59	1.98 ± 0.12 ^fg^	3.05 ± 0.12 ^l^
*A. kunkeei* BIII60	1.28 ± 0.18^ij^	3.36 ± 0.24^ijkl^
*A. kunkeei* BVI14	1.41 ± 0.13^i^	2.99 ± 0.29 ^l^
*A. kunkeei* B17	2.41 ± 0.14^de^	8.32 ± 0.28^b^
*A. kunkeei* B23I	3.51 ± 0.10^a^	4.21 ± 0.22^f^
*A. kunkeei* B7	1.81 ± 0.19^gh^	3.06 ± 0.12 l
*A. kunkeei* BV61	1.87 ± 0.14^gh^	3.76 ± 0.36^fghijk^
*A. kunkeei* BV20	2.05 ± 0.13 ^fg^	4.16 ± 0.28 ^fg^
*A. kunkeei* BVI52	2.79 ± 0.08^bc^	3.84 ± 0.24^fghi^
*A. kunkeei* B4I	1.89 ± 0.07^gh^	3.11 ± 0.26^jkl^
*A. kunkeei* PLB29	1.42 ± 0.10^i^	3.65 ± 0.21^ghij^
*A. kunkeei* PLA21	2.92 ± 0.15^bc^	3.85 ± 0.14^fgh^
*A. kunkeei* PFA3	1.75 ± 0.21^gh^	7.39 ± 0.38^c^
*A. kunkeei* PLB20	1.86 ± 0.22^gh^	3.50 ± 0.25^hijk^
*A. kunkeei* PLB 34	2.57 ± 0.19^cde^	5.64 ± 0.36^d^
*A. kunkeei* PL24	1.50 ± 0.24^hi^	1.47 ± 0.17 ^m^
*A. kunkeei* PF16	2.66 ± 0.28^bcde^	3.43 ± 0.22^hijkl^
*A. kunkeei* PF6	3.20 ± 0.18^bc^	5.18 ± 0.31^de^
*A. kunkeei* BEE4	3.24 ± 0.23^ab^	7.46 ± 0.27^c^
*Fructobacillus fructosus* MBIII5	3.02 ± 0.15^bc^	5.30 ± 0.33^d^
*Fructo. fructosus* B5	2.22 ± 0.17^ef^	3.83 ± 0.28^fghi^
*Fructo. fructosus* MBIII2	2.26 ± 0.19^ef^	3.07 ± 0.25^kl^
*Fructo. fructosus* B1	1.73 ± 0.22^gh^	4.78 ± 0.24^e^
*Fructo. fructosus* PL21	2.82 ± 0.17^bc^	5.32 ± 0.32^d^
*Fructo. fructosus* PFA23	0.96 ± 0.15^jk^	3.29 ± 0.31^ijkl^
*Lactiplantibacillus plantarum* DC400	0.28 ± 0.14 ^l^	0.22 ± 0.08^n^
*Fructilactobacillus sanfranciscensis* SD8	0.88 ± 0.08 ^k^	13.76 ± 0.39^a^

Fructose consumption was determined during growth of bacterial strains in FGYP broth at 30 °C for 24 h

*Kinetics were modelled according to the Gompertz equation as modified by Zwietering et al. [14]: *μ*_*max*_ is maximum consumption rate (expressed as g l^−1^ h^−1^), *λ* is the length of the lag phase expressed in hours

^a−n^Values in the same column with different superscript letters are significantly different (P < 0.05)

### Mannitol consumption by FLAB into a mannitol-based media

**Fig. 1:**
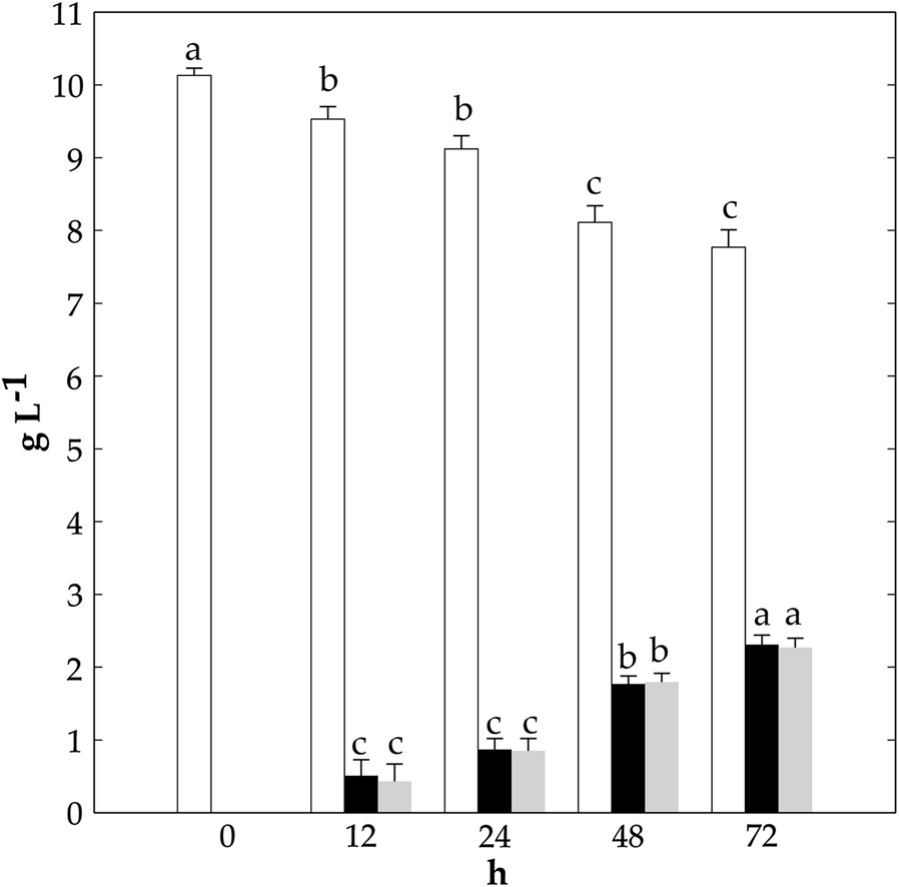
Mannitol (white bars) and lactic (black bars) and acetic (grey bars) acids level (g L^−1^). *Fructobacillus fructosus* MBIII5 was grown in Mannitol Yeast extract Peptone (MYP) broth at 30 °C for 72 h under aerobic condition in shaking (200 rpm) flasks with baffles. For each compound, bars with different superscript letters (a–c) are significantly different (P < 0.05)

Aiming at selecting the best performing FLAB strains to metabolize mannitol, they were grown on Mannitol Yeast extract Peptone (MYP) agar supplemented with CaCO_3_, containing mannitol as the only carbon source. Mannitol consumption was evaluated by measuring the diameter of the clearance zone surrounding the colonies. Only for six strains, a clearance zone was observed. *Fructo. fructosus* MBIII5 showed the widest (P < 0.05) halo (2.6 ± 0.3 cm), followed by *Fructo. fructosus* MBIII2 (1.6 ± 0.4 cm) and B5 (1.4 ± 0.4 cm), and *A. kunkeei* B7 (1.3 ± 0.7 cm). The less extensive halos were found for *Fructo. fructosus* PL21 (0.4 ± 0.3 cm) and PFA23 (0.4 ± 0.4 cm). Based on the highest metabolic activity of *Fructo. fructosus* MBIII5 on MYP agar compared to the other strains, mannitol consumption by *Fructo. fructosus* MBIII5 was further investigated during growth into MYP broth containing mannitol (10 g L^−1^) as the only carbon source (Fig. 1). During 24 h of incubation, this strain metabolized mannitol (1.02 ± 0.10 g L-1) to lactic (0.87 ± 0.15 g L-1) and acetic acids (0.85 ± 0.17 g L-1). Mannitol was further metabolized after 48 (2.03 ± 0.14 g L-1) and 72 h (2.36 ± 0.15 g L-1) of incubation (Fig. 1).

### Growth and acidification performances of the selected FLAB strains under sourdough conditions

Based on the kinetics of fructose consumption that showed the highest value of *μ*_*max*_ (3.51 ± 0.10 g L^−1^ h^−1^), *A. kunkeei* B23I was selected as starter for sourdough fermentation (DY 160). *Fructo. fructosus* MBIII5 was also selected for its capability to use mannitol as carbon source. *L. plantarum* DC400 and *F. sanfranciscensis* SD8 were used as control starters. A control dough (DY 160) was prepared only with durum wheat flour and sterile tap water, without bacterial inoculum (unstarted dough) and incubated for 24 h. From an initial cell density of ca. 7 Log CFU g^−1^, both selected FLAB strains grew by ca. 1.8 logarithmic cycles after 12 h at 30 °C. Cell density remained almost stable until 24 h. *L. plantarum* DC400 led to an increase of ca. 2 and 2.5 log cycles after 12 and 24 h, respectively. *F. sanfranciscensis* SD8 grew by ca. 1.6 Log cycles after 12 h and by ca. 2 Log cycles after 24 h. After 24 of incubation, the cell density of lactic acid bacteria into the unstarted dough was ca. 3 Log CFU g^−1^.

The initial pH of the dough was 6.2 ± 0.02. After 12 h, the lowest (P < 0.05) value of pH was found for *L. plantarum* DC400 (4.08 ± 0.03), followed by *A. kunkeei* B23I (4.86 ± 0.01), *Fructo. fructosus* MBIII5 (5.04 ± 0.02) and *F. sanfranciscensis* SD8 (5.01 ± 0.02). Values of pH dropped further throughout the fermentation and reached the final values of 4.16 ‒ 4.11 with *A. kunkeei* B23I, *Fructo. fructosus* MBIII5, and *F. sanfranciscensis* SD8. The lowest (P < 0.05) value was found for *L. plantarum* DC400 (3.78 ± 0.03). The pH values of the unstarted dough ranged from 6.2 to 5.75.

### Total fructans, fructose, glucose, and mannitol levels in fermented doughs

**Fig. 2:**
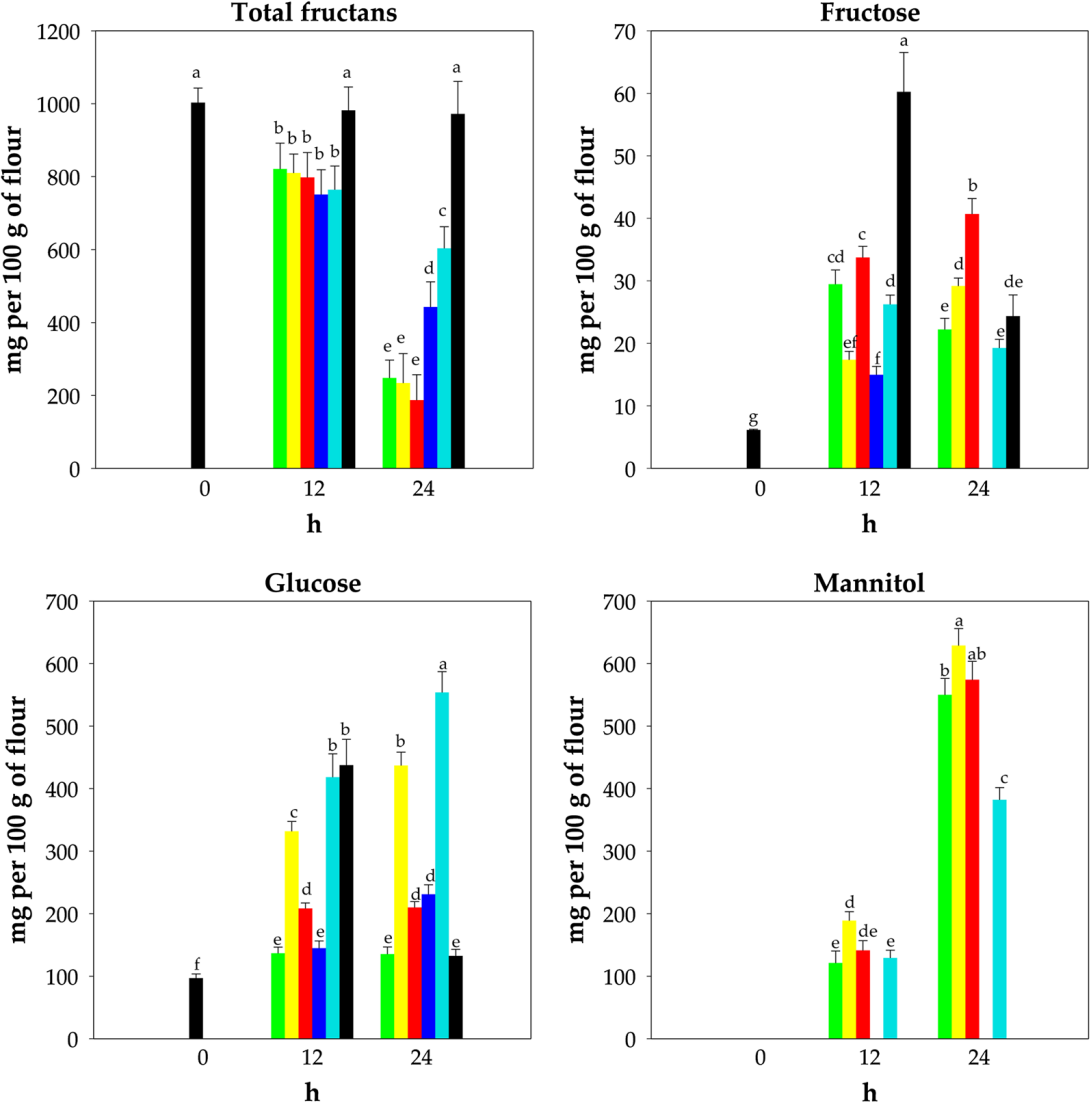
Total fructans, fructose, glucose, and mannitol level (mg per 100 g of flour) in doughs. Wheat doughs (DY 160) were fermented with *Apilactobacillus kunkeei* B23I (green bars), *Fructobacillus fructosus* MBIII5 (yellow bars), *A. kunkeei* B23I + *Fructo. fructosus* MBIII5 (red bars), *Lactiplantibacillus plantarum* DC400 (blue bars) or *Fructilactobacillus sanfranciscensis* SD8 (cyan bars) at 30 °C for 24 h. A control dough without bacterial inoculum was incubated under the same conditions (black bars). Bars with different superscript letters (a–g) are significant different (P < 0.05)

*A. kunkeei* B23I and *Fructo. fructosus* MBIII5 were used both as single starters and as binary combination to ferment wheat flour doughs (DY 160) for 24 h at 30 °C. Doughs fermented by *L. plantarum* DC400 or *F. sanfranciscensis* SD8 were also analyzed. A dough without bacterial inoculums was used as control (unstarted dough). Total fructans, fructose, glucose, and mannitol were monitored after 12 and 24 h of fermentation. The initial concentration of total fructans was 1003 ± 40 mg per 100 g of flour. After 12 h only a slow decrease of fructans (18-25%) was found in all fermented samples. After 24 h of fermentation with the two selected FLAB strains, the concentration of fructans significantly (P < 0.05) decreased by 75-81% (Fig. 2). The drop (P < 0.05) observed with *L. plantarum* DC400 and *F. sanfranciscensis* SD8 after the same time was of ca. 56% and 40%, respectively (Fig. 2). No significant (P > 0.05) change was found for the unstarted dough (Fig. 2).

Compared to the initial concentration of fructose (6.2 ± 0.1 mg per 100 g of flour), an increasing release (P < 0.05) was observed during fermentation with the selected strains (Fig. 2). After 24 h of fermentation by selected FLAB, the level of fructose increased to 22.2‒40.7 mg per 100 g of flour (Fig. 2). Only *L. plantarum* DC400 was able to fully deplete fructose, whereas with *F. sanfranciscensis* SD8 the residual fructose was 19.3 ± 1.4 mg per 100 g of flour (Fig. 2). Fructose in excess of glucose was not found in any sample (Fig. 2). Mannitol was mainly released in doughs fermented with FLAB strains and, to a lesser extent, with *F. sanfranciscensis* SD8 (Fig. 2). After 24 h, the highest amount of mannitol was detectable with *Fructo. fructosus* MBIII5 (629.1 ± 27.2 mg per 100 g of flour) (Fig. 2). Mannitol was never detected in the dough fermented with *L. plantarum* DC400 and into the unstarted dough (Fig. 2).

### Protocol set up and validation

Preliminary trials were carried out to define the best conditions in order to achieve the efficient enzymatic hydrolysis of fructans in wheat doughs (DY 160). The enzymatic treatment with invertase (100 U g^−1^ of substrates) at 35 °C for 3 h was suitable to reach the hydrolysis of most of fructans (ca. 80%). Combinations of FLAB and enzymatic treatment were performed in order to fully degrade FODMAPs in firm (DY 160) and liquid (DY 280) wheat doughs. Accordingly to the defined protocol, doughs were made by mixing invertase (100 U g^−1^ of substrates) and inoculating (ca. 8 Log CFU g^−1^) with single starter, composed by *A. kunkeei* B23I (Treatment 1, T1) or *Fructo. fructosus* MBIII5 (Treatment 2, T2), or mixed starters consisting of *A. kunkeei* B23I and *Fructo. fructosus* MBIII5 (Treatment 3, T3). A further dough was made and used as a control, which included the invertase and a mixed starter comprising *L. plantarum* DC400 and *F. sanfranciscensis* SD8 (Control treatment, CT). Doughs were fermented at 35 °C for 3 h. Liquid doughs were incubated under aerobic condition in shaking (200 rpm) flasks with baffles.

**Fig. 3:**
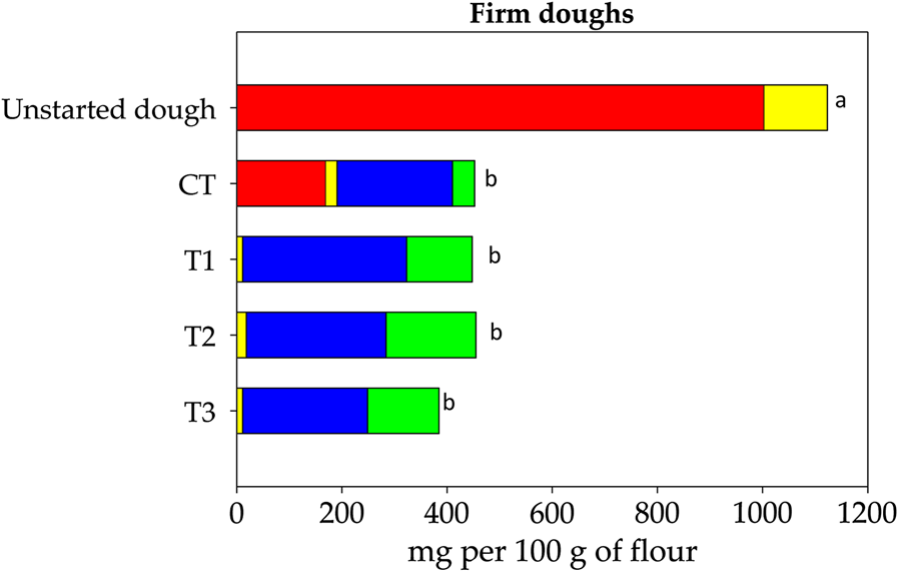
FODMAPs (mg per 100 g of flour) in firm (DY 160) doughs. Wheat doughs were added with invertase (EC 3.2.1.26) and fermented at 35 °C for 3 h with *Apilactobacillus kunkeei* B23I (Treatment 1, T1), or *Fructobacillus fructosus* MBIII5 (Treatment 2, T2), or with a mixed starter composed by *A. kunkeei* B23I and *Fructo. fructosus* MBIII5 (Treatment 3, T3), or *Lactiplantibacillus plantarum* DC400 and *Fructilactobacillus sanfranciscensis* SD8 (Control treatment, CT). Unstarted dough was also analyzed. Total fructans, red bars; galactosyl-sucrose oligosaccharides, yellow bars; fructose in excess of glucose, blue bars; mannitol, green bars. Bars with different superscript letters (a–d) are significant different (P < 0.05)

**Fig. 4:**
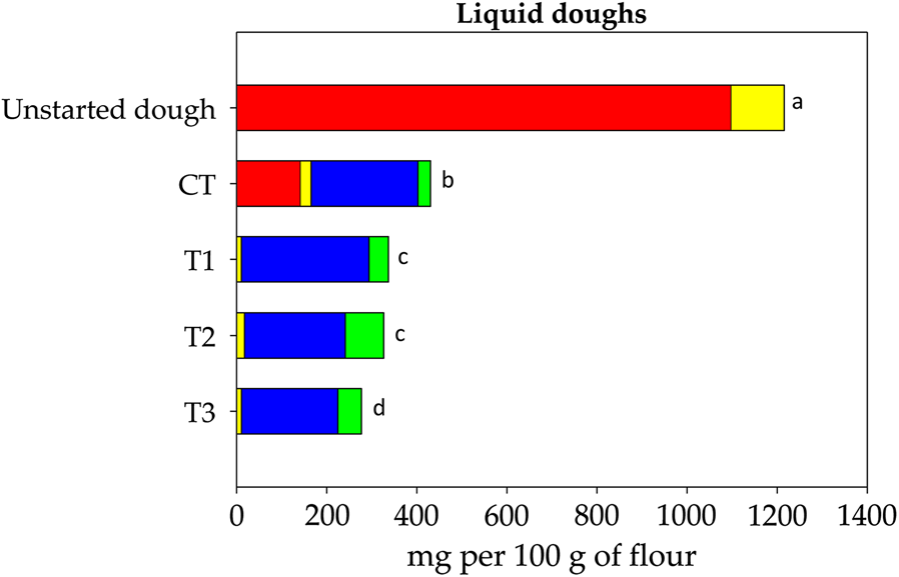
FODMAPs (mg per 100 g of flour) in liquid (DY 280) doughs. Wheat doughs were added with invertase (EC 3.2.1.26) and fermented at 35 °C for 3 h under aerobic condition (in shaking [200 rpm] flasks with baffles) with *Apilactobacillus kunkeei* B23I (Treatment 1, T1), or *Fructobacillus fructosus* MBIII5 (Treatment 2, T2), or with a mixed starter composed by *A. kunkeei* B23I and *Fructo. fructosus* MBIII5 (Treatment 3, T3), or *Lactiplantibacillus plantarum* DC400 and *Fructilactobacillus sanfranciscensis* SD8 (Control treatment, CT). Unstarted dough was also analyzed. Total fructans, red bars; galactosyl-sucrose oligosaccharides, yellow bars; fructose in excess of glucose, blue bars; mannitol, green bars. Bars with different superscript letters (a-d) are significant different (P < 0.05)

As shown in Figs. 3 and 4, the effect of treatments on FODMAPs level was mainly starter dependent, even though process parameters such DY and incubation conditions also contributed. Fructans were completely depleted in doughs fermented by FLAB (treatments T1, T2, and T3) both under firm and liquid conditions. On the contrary, only 83‒87% of fructans were hydrolyzed with CT (Figs. 3 and 4). Fructose in excess of glucose ranged from 214.2 to 311.9 mg per 100 g of flour in doughs fermented by FLAB, and from 219.5 to 237.3 mg per 100 g of flour in CT (Figs. 3 and 4). GOS underwent a reduction of 80-91% depending on the treatment (Figs. 3 and 4). Mannitol was mainly detectable in firm doughs subjected to T1, T2 and T3 (124.7‒171.1 mg per 100 g of flour) (Fig. 3). Significantly lower (P < 0.05) amount of mannitol were detectable in liquid doughs under the same treatments (43.2‒85.2 mg per 100 g of flour) (Fig. 4). Mannitol levels in doughs subjected to CT were 42.2 and 28.0 mg per 100 g of flour under firm and liquid conditions, respectively (Figs. 3 and 4). Considering the total amount of FODMAPs, no significant differences (P > 0.05) were found between T1, T2, T3 and CT in firm doughs. On the contrary, fermentation of liquid doughs with FLAB led to lower (P < 0.05) levels of FODMAPs compared to CT. Overall, the lowest (P < 0.05) amount of FODMAPs (277.8 mg per 100 g of flour) was reached with T3 under liquid conditions (Fig. 4).

## Discussion

Graminan-type fructans are the main FODMAPs in wheat-based products, and their content may be reduced via bioprocessing with endogenous enzymes and/or microbial fermentation [1, 2, 9]. Sourdough fermentation should allow diminishing FODMAP levels [9, 15]. The extent of reduction is dependent on the sourdough dosage, time of fermentation, type of flour, and, especially, the microbiota. Yeasts and few lactic acid bacteria species are able to hydrolyze fructans. During bioprocessing treatment on fructans, side reactions might lead to new FODMAPs. For instance, fructanase treatment releases large amounts of fructose, which in turn may be converted into mannitol throughout fermentation [9]. Therefore, our study investigated the complementary capability of targeted commercial enzymes and metabolically strictly FLAB to hydrolyze fructans and deplete fructose during wheat dough fermentation. FLAB prefer fructose as a growth substrate, because of its dual function as carbon source and external electron acceptor to achieve the NAD^+^ co-factor regeneration by the reduction of fructose to mannitol. Their growth on glucose depends on the availability of an additional electron acceptor, like fructose or oxygen [16–24]. Despite the evidence of their preferential consumption of fructose, little information is available on the biokinetics of FLAB during the growth in complex media or under food-like conditions. Our approach combined growth assays in synthetic media containing more than one sugar as substrate and fermentation trials in wheat doughs. During growth into a medium containing both fructose and glucose, FLAB strains displayed higher fructose consumption rate compared to conventional sourdough lactic acid bacteria such as *L. plantarum* and *F. sanfranciscensis*. Both fructophilic species *A. kunkeei* and *Fructo. fructosus* showed the same metabolic behavior although differences were found at strain level, with *A. kunkeei* B23I as the best performing. Fructose metabolism by FLAB was faster than glucose, regardless of the presence of oxygen, which can be used as external electron acceptor in alternative to fructose [16–24]. These preliminary findings suggested that fructose transport is privileged in FLAB over glucose, and supports their potential role in depletion of fructose resulting from fructans hydrolysis. Accordingly to the literature, we observed a very low fructose consumption rate by conventional sourdough lattobacilli used as controls [25]. In fact, fructose utilization by homo-fermentative and facultative hetero-fermentative species generally take place only after glucose depletion or for cell maintenance, whereas hetero-fermentative lactobacilli use fructose mostly as an electron acceptor. For instance, most of *F. sanfranciscensis* strains are unable to use fructose as carbon source, but generally display mannitol dehydrogenase activity and reduce fructose to mannitol [25, 26].

Because of the FLAB requirement to maintain their redox balance by reducing fructose into mannitol, this latter might accumulate during wheat dough fermentation. Therefore, we investigated the catabolism of mannitol with the goal of its reuse by FLAB. Only *A. kunkeei* B7 and five *Fructo. fructosus* strains were able to degrade mannitol, although its consumption rate was lower compared to fructose and glucose. Mannitol metabolism has been previously described in homo-fermentative lactobacilli as mediated by a mannitol-specific PTS system and a mannitol-1-phosphate-dehydrogenase [27, 28]. While FLAB have been previously shown to metabolize mannitol [16, 24], in this bacterial group the underling pathway and regulatory mechanism have not been rationalized. Based on our findings, this pathway is differently expressed by FLAB. *Fructo. fructosus* MBIII5 was the most active strain in mannitol consumption, leading to lactic and acetic acids as products.

Our mechanistic approach allowed the selection of two FLAB strains as starter candidates for low FODMAPs baking. *A. kunkeei* B23I was chosen for its high fructose consumption rate and *Fructo. fructosus* MBIII5 was selected for its ability to use mannitol as carbon source. Under sourdough condition, these two strains showed growth and acidification performances almost comparable to those of *L. plantarum* and *F. sanfranciscensis* strains. Anyway, the assessment of the degradation and consumption of saccharides during wheat dough fermentation revealed different metabolic features among different strains. Higher fructans breakdown occurred in FLAB inoculated doughs. Studies describing fructans degradation by FLAB are lacking. Only Prückler et al. [29] previously reported fructans breakdown in wheat bran fermented by *Fructo. fructosus*, but the same extent was observable with *F. sanfranciscensis*. Metabolism of fructans in lactobacilli is mediated by ATP-Binding-Cassette transport system, phosphoenolpyruvate-dependent phosphotranferase transport system (β-glucoside family), and intracellular fructosidases [30]. In wheat flour, a large fraction of fructans exhibits a degree of polymerization (DP) ranging from 4 and 19, and the hydrolysis requires extracellular fructanases, which are infrequent in lactobacilli. Extracellular fructanases were characterized in *Lactobacillus paracasei*, and *Lactobacillus crispatus* [31, 32], and analogous DNA sequence of extracellular fructanases were identified in genome of few other species [9]. The different enzymatic pathways for fructans utilization by lactobacilli determine their abilities to degrade low- or high-DP fructans. Intracellular fructosidases limit the ability of lactobacilli to use only low-DP fructans [9, 30]. Based on our evidences concerning the high hydrolysis capability by FLAB, their fructans utilization pathways deserve further investigation with the prospect of identifying enzymatic activities uncommon in lactobacilli. During wheat dough fermentation, the high fructose consumption rate by FLAB was confirmed, although the consumption was partly masked due to the release of free fructose during the decomposition of fructans, sucrose, and GOS. Both *A. kunkeei* B23I and *Fructo. fructosus* MBIII5 led to an increase in mannitol levels, despite the ability of *Fructo. fructosus* MBIII5 to degrade mannitol was shown into a synthetic medium. It is not unlikely that reutilization of mannitol would occur only after carbohydrate sources exhaustion and mannitol accumulation, due to induction of the mannitol transport system.

Preliminary trials allowed to develop a novel fermentation protocol exploiting the complementary capability of commercial invertase and selected FLAB strains for low FODMAPs baking. Fermentations were carried out using *A. kunkeei* B23I and *Fructo. fructosus* MBIII5 as single or binary starter both under firm- and liquid-dough conditions. Overall, the use of binary starter to ferment liquid dough led to the lowest amount of FODMAPs (277.8 mg per 100 g of flour). Based on the proposed threshold value for single FODMAPs compound [7, 33], the residual level in bread prepared according to our FLAB-based protocol was low enough to minimize symptom induction in FODMAPs sensitive people but guarantying a fiber intake comparable to regular bread. A low-FODMAPs rye bread with a total fructans content of 0.3 g per 100 g of brad was previously shown to reduce symptoms and gastrointestinal gas accumulation in IBS patients [33]. Our protocol did not exert the same positive effect on the total FODMAPs under firm-dough conditions, mainly due to the high accumulation of mannitol. Likely, liquid dough fermentation under aerobic condition allowed higher oxygenation than in firm dough, resulting in lower mannitol production [28]. Secondarily, oxygen may have supported mannitol catabolism by *Fructo. fructosus* MBIII5, as the requirement for an external electron acceptor has been previously described for other lactobacilli during mannitol fermentation [34].

## Conclusions

The results of our pioneering study clearly demonstrated the potential of selected strictly FLAB to strongly reduce FODMAPs in wheat dough, particularly under liquid-dough and high oxygenation conditions. Although the best performances have been reached applying our protocol to liquid doughs, we have to point that the industrial demand for more efficient, controllable, largescale fermentation processes led to prefer type II sourdoughs, which are pumpable (semi-fluid) and consequently easy to handle. Our standardized protocol is also suitable for type III sourdough preparations.

Further investigation should be performed to evaluate potential synergistic interactions between FLAB and yeasts. Yeasts such as *Saccharomyces cerevisiae* and *Kluyveromyces marxianus* have proved their ability to degrade fructans [2] and therefore might contribute to reduce FODMAP levels in a complementary way with FLAB and commercial enzymes. Since FLAB have been found in association with yeasts in several ecological niches, we hypothesized the absence of antagonistic relationships between these two microbial groups, although specific negative interactions at strain or species level may not be excluded [35, 36].

Clinical trials will need to confirm the suitability for patients with IBS or non-celiac wheat sensitivity of wheat breads obtained according to our protocol.

## Methods

### Microorganisms and culture conditions

We used for fermentation 25 strains of strictly FLAB (Table 2). Previously, the strains isolation was from bee-gut (*Apis mellifera L.*) and ivy (*Hedera helix* L.) pollen [16, 35]. *Lactiplantibacillus plantarum* DC400 and *Fructilactobacillus sanfranciscensis* SD8 were the control strains (without preference of fructose over glucose as growth substrate), representing the conventional species of lactic acid bacteria mostly involved in sourdough fermentation [37]. All the strains belong to the Culture Collection of the Department of Soil, Plant and Food Science, University of Bari Aldo Moro (Bari, Italy), whose identification was by partial sequencing of the *16S rRNA* and *recA* genes. Cultures were maintained as stocks in 15% (v v^−1^) glycerol at -80 °C and routinely propagated. FLAB strains were grown in Fructose Yeast extract Peptone (FYP) broth (10 g d-fructose, 10 g yeast extract, 5 g polypeptone, 2 g sodium acetate, 0.5 g Tween 80, 0.2 g MgSO_4_·7H_2_O, 0.01 g MnSO_4_·4H_2_O, 0.01 g FeSO_4_·7H_2_O, 0.01 g NaCl per liter of distilled water [pH 6.8]) at 30 °C until the late exponential growth phase was reached (ca. 18 h) [17]. *L. plantarum* DC400 and *F. sanfranciscensis* SD8 were grown in Sour Dough Bacteria (SDB) broth at 30 °C until the late exponential growth phase was reached (ca. 18 h) [38].

**Table 2:** Strictly FLAB strains (n = 25) used in this study

Strain	Source	Reference
*Apilactobacillus kunkeei* BIII59, BIII60, BVI14, B17, B23I, B7, BV61, BV20, BVI52, B4I	*Apis mellifera L.* bee-gut	16
*A. kunkeei* BEE4	*A. mellifera L.* bee-gut	Filannino et al., unpublished data
*A. kunkeei* PLB29, PLA21, PFA3, PLB20, PLB 34, PL24, PF16, PF6	*Hedera helix* L. pollen	35
*Fructobacillus fructosus* MBIII5, B5, MBIII2, B1	*A. mellifera L.* bee-gut	16
*Fructo. fructosus* PL21, PFA23	*H. helix* L. pollen	35

### Fructose consumption rate by FLAB into a fructose-glucose based medium

All strains were screened for the determination of fructose consumption rate by using a fructose-glucose based medium. Cells were cultivated in FYP broth until the late exponential growth phase was reached (ca. 18 h), harvested by centrifugation (10,000×*g* for 10 min at 4 °C), washed twice in 50 mM phosphate buffer, pH 7.0, and re-suspended at the initial cell density of ca. 7 Log CFU mL^−1^ into a Fructose Glucose Yeast extract Peptone (FGYP) broth (identical to FYP broth but added with glucose, 1% w v^−1^). Then, cells were incubated aerobically at 30 °C for 24 h. Supernatants were recovered from FGYP cultures every 2 h for 24 h, centrifuged at (10,000×*g* for 10 min at 4 °C), filtered through 0.22-μm-pore-size filter (Millipore), and used to determine glucose and fructose by high-performance liquid chromatography (HPLC). An Äkta purifier system (GE Healthcare) equipped with an Aminex HPX-87H column (ion exclusion; Bio-Rad), and a PerkinElmer 200a refractive index detector (PerkinElmer, Waltham, MA) operating at 32 °C were used. Elution was at 60 °C with a flow rate of 0.4 ml min^−1^, and 10 mmol l^−1^ H_2_SO_4_ was used as mobile phase [39]. Commercial standards (Sigma-Aldrich; Merck KGaA, Darmstadt, Germany) allowed the identification and quantification of each compounds. Kinetics of fructose consumption were determined and modeled according to the Gompertz equation as modified by Zwietering et al. [14]:

$$y\, = \,k\, + \,D\,\exp \left( {\text{ - }\exp \left( {\left( {\left( {\mu_{\hbox{max} } \times2.7182} \right)\left( {\lambda \text{ - }t} \right)\text{/}D} \right) + \,1} \right)} \right).$$

where k is the initial level of fructose (expressed as g l^−1^), *D* is the highest amount of fructose consumed (expressed as g l^−1^) at the stationary phase, *μ*_*max*_ is maximum consumption rate (expressed as g l^−1^ h^−1^), *λ* is the length of the lag phase expressed in hours, and *t* is the time. Experimental data were modelled by the non-linear regression procedure of the Statistica 8.0 software (Statsoft, Tulsa, USA).

### Mannitol consumption by FLAB in mannitol-based media

FLAB strains were sub-cultured in modified FYP broth containing fructose (0.5% w v^−1^) and mannitol (1% w v^−1^) as carbon sources. Sub-cultured cells were harvested, washed as described above, and inoculated on Mannitol Yeast extract Peptone (MYP) agar (identical to FYP medium but fructose was replaced by mannitol) supplemented with 0.5% CaCO_3_ (w v^−1^). Plates were incubated at 30 °C for 48 h. Mannitol consumption was evaluated by measuring the size of the clearance zone surrounding the colonies, which indirectly indicates the hydrolysis of CaCO_3_ reacting with organic acids synthesized by bacteria.

Mannitol consumption and deriving metabolites were also investigated during FLAB growth in MYP broth. Cells were cultivated in FYP broth until the late exponential growth phase was reached (ca. 18 h), harvested as described above, and re-suspended at the initial cell density of ca. 7 Log CFU mL^−1^ into a MYP broth. Cells were cultured at 30 °C for 72 h under aerobic condition in shaking (200 rpm) flasks with baffles. Organic acids and mannitol concentrations were determined by HPLC as described above, using external standards (Sigma-Aldrich). The pH was measured by a Crison pH-meter (Model 507, Crison, Milan, Italy).

### Model-sourdough fermentation

Commercial durum wheat (*Triticum durum*) flour was purchased from a local market in Italy. Gross composition was as follows: moisture, 12.6 ± 0.42%; protein (N × 5.7), 11.9 ± 0.01% of dry matter (d.m.); total carbohydrates, 70.9 ± 0.3% of d.m.; fat 1.0 ± 0.01% of d.m. Flour (187.5 g) and tap water (112.5 ml) were used to produce 300 g of dough (dough yield [dough weight × 100/flour weight], 160) with a continuous high-speed mixer (60 × g, dough mixing time of 5 min) (Chopin & Co., Boulogne, Seine, France).

Pure culture of selected *A. kunkeei* B23I and *Fructo. fructosus* MBIII5 strains were used singly or as mixed starters. The mixed starter consisted of binary combinations including two bacterial strains inoculated at the same cell density (ca. 7 Log CFU g^−1^). Positive interactions among strains or species of lactic acid bacteria have been described by several studies [40]. Thus, in this study we proposed the combination of *A. kunkeei* B23I with *Fructo. fructosus* MBIII5 to exploit complementarily their metabolic traits, which were highlighted during the preliminary trials. *L. plantarum* DC400 and *F. sanfranciscensis* SD8 were used as control starters. Cells were cultivated in FYP or SDB broths and inoculated into the dough at the initial cell density of ca. 7 Log CFU g^−1^, as described above. A control dough was prepared with durum wheat flour and sterile tap water, without bacterial inoculums (unstarted dough). Doughs were incubated for 24 h at 30 °C. Acidification and bacterial growth were monitored every 12 h for 72 h. The pH value of doughs was determined by a pH-meter with a food penetration probe. Microbiological analyses were carried out on 10 g of dough homogenized with 90 ml of sterile peptone water (peptone 1% wt/vol and NaCl 0.9% wt/vol) solution. Presumptive lactic acid bacteria were enumerated by plating on FYP agar or SDB agar. Media were supplemented with cycloheximide (0.1 g L^−1^). Plates were incubated at 30 °C for 48 h.

### Determination of total fructans, fructose, glucose, and mannitol levels in fermented doughs

Levels of total fructans, fructose, glucose, and mannitol were monitored every 12 h during fermentation. Samples were freeze-dried and grinded before analyses.

Glucose, fructose and mannitol were quantified by HPLC as described above. Prior the analysis, 10 mL of perchloric acid (5%, v v^−1^) were added to 10 g of sample as precipitating agent. Then, the suspension was kept overnight at 4 °C, centrifuged at 10,000×*g* for 10 min, and filtered through a 0.22-mm pore size filter.

Total fructans were measured using the Fructan Assay Kit (Megazyme International Ireland Ltd) as per manufacturer’s instructions. The extraction process was carried out as described by Muir et al. [41], with few changes. One g of freeze-dried dough was milled and mixed with 40 ml of hot distilled water (80 °C), and stirred on a hot-plate (80 °C) for 15 min, until the sample is completely dispersed. Then, sample was cooled at room temperature and the volume was adjusted to 50 ml with distilled water.

### Enzymes

Endo-inulinase from *Aspergillus niger* (EC 3.2.1.7), exo-inulinase from *A. niger* (EC 3.2.1.80), and invertase (fructofuranosidase) from yeasts (EC 3.2.1.26) were purchased from Megazyme International Ireland Ltd. The enzyme activities were investigated during preliminary trials carried out on wheat flour doughs (DY 160) under different concentrations (10–100 U g^−1^ of substrates), temperatures (30-37 °C), and time of incubation (2–4 h) in order to define the best conditions of use. Fructans hydrolysis was monitored by HPLC analysis and the Fructan Assay Kit (Megazyme International Ireland Ltd) as described above.

### Protocol set up and validation

Based on the preliminary experiment results, further fermentation trials were carried out on firm (DY, 160) and liquid (DY, 280) wheat doughs. Doughs were mixed with invertase (100 U g^−1^ of substrates) and inoculated at a cell density of ca. 8 Log CFU g^−1^ with a starter composed by *A. kunkeei* B23I (Treatment 1, T1) or *Fructo. fructosus* MBIII5 (Treatment 2, T2), or a mixed starter composed by a combination of *A. kunkeei* B23I and *Fructo. fructosus* MBIII5 (Treatment 3, T3). A further condition was used as a control combining the enzymatic treatment with a mixed starter composed of *L. plantarum* DC400 and *F. sanfranciscensis* SD8 (Control treatment, CT). The mixed starters consisted of binary combinations of two bacterial strains both inoculated at a cell density of ca. 8 Log CFU g^−1^. Doughs were fermented at 35 °C for 3 h. Liquid doughs were incubated under aerobic condition in shaking (200 rpm) flasks with baffles.

Levels of total fructans, fructose, glucose, and mannitol were determined as described above. GOS were measured using an enzymatically based assay kit (Raffinose/D-Galactose Assay Kit, Megazyme International Ireland Ltd), according to the manufacturer’s instructions.

### Statistical analysis

All the fermentations were in triplicate and samples underwent analysis in triplicate. Data underwent to one-way ANOVA by Statistica for Windows (Statistica7.0 per Windows). Tukey’s test was used to determine significant differences among means at an error probability of 5% (P < 0.05).

## Data Availability

The data sets supporting the conclusions of this article are included within the article.

## Abbreviations

FODMAPs: Fermentable oligosaccharides, disaccharides, monosaccharides, and polyols
FLAB: Fructophilic lactic acid bacteria
GOS: Galacto-oligosaccharides
IBS: Irritable bowel syndrome
FYP: Fructose Yeast extract Peptone
FGYP: Fructose Glucose Yeast extract Peptone
MYP: Mannitol Yeast extract Peptone
T1: Treatment 1
T2: Treatment 2
T3: Treatment 3
CT: Control treatment
